# Suppressor Mutations in Type II Secretion Mutants of Vibrio cholerae: Inactivation of the VesC Protease

**DOI:** 10.1128/mSphere.01125-20

**Published:** 2020-12-16

**Authors:** Chelsea S. Rule, Young-Jun Park, Jaclyn R. Delarosa, Stewart Turley, Wim G. J. Hol, Sarah McColm, Colby Gura, Frank DiMaio, Konstantin V. Korotkov, Maria Sandkvist

**Affiliations:** a Department of Microbiology and Immunology, University of Michigan Medical School, Ann Arbor, Michigan, USA; b Department of Biochemistry, University of Washington, Seattle, Washington, USA; c Department of Molecular and Cellular Biochemistry, University of Kentucky, Lexington, Kentucky, USA; University of Iowa

**Keywords:** *Vibrio cholerae*, type II secretion system, serine protease, suppressor, protein structure

## Abstract

The type II secretion system (T2SS) is a conserved transport pathway responsible for the secretion of a range of virulence factors by many pathogens, including Vibrio cholerae. Disruption of the T2SS genes in V. cholerae results in loss of secretion, changes in cell envelope function, and growth defects. While T2SS mutants are viable, high-throughput genomic analyses have listed these genes among essential genes. To investigate whether secondary mutations arise as a consequence of T2SS inactivation, we sequenced the genomes of six V. cholerae T2SS mutants with deletions or insertions in either the *epsG*, *epsL*, or *epsM* genes and identified secondary mutations in all mutants. Two of the six T2SS mutants contain distinct mutations in the gene encoding the T2SS-secreted protease VesC. Other mutations were found in genes coding for V. cholerae cell envelope proteins. Subsequent sequence analysis of the *vesC* gene in 92 additional T2SS mutant isolates identified another 19 unique mutations including insertions or deletions, sequence duplications, and single-nucleotide changes resulting in amino acid substitutions in the VesC protein. Analysis of VesC variants and the X-ray crystallographic structure of wild-type VesC suggested that all mutations lead to loss of VesC production and/or function. One possible mechanism by which V. cholerae T2SS mutagenesis can be tolerated is through selection of *vesC*-inactivating mutations, which may, in part, suppress cell envelope damage, establishing permissive conditions for the disruption of the T2SS. Other mutations may have been acquired in genes encoding essential cell envelope proteins to prevent proteolysis by VesC.

**IMPORTANCE** Genome-wide transposon mutagenesis has identified the genes encoding the T2SS in Vibrio cholerae as essential for viability, but the reason for this is unclear. Mutants with deletions or insertions in these genes can be isolated, suggesting that they have acquired secondary mutations that suppress their growth defect. Through whole-genome sequencing and phenotypic analysis of T2SS mutants, we show that one means by which the growth defect can be suppressed is through mutations in the gene encoding the T2SS substrate VesC. VesC homologues are present in other *Vibrio* species and close relatives, and this may be why inactivation of the T2SS in species such as Vibrio vulnificus, *Vibrio* sp. strain 60, and Aeromonas hydrophila also results in a pleiotropic effect on their outer membrane assembly and integrity.

## Abstract (precis)

Genome-wide transposon mutagenesis has identified the genes encoding the T2SS in Vibrio cholerae as essential for viability, but the reason for this is unclear. Mutants with deletions or insertions in these genes can be isolated, suggesting that they have acquired secondary mutations that suppress their growth defect.

## INTRODUCTION

Vibrio cholerae is a Gram-negative bacterial pathogen and the causative agent of the disease cholera. Upon colonization of the human small intestine, V. cholerae infection causes profuse diarrhea, which can lead to rapid dehydration without oral rehydration therapy (1, 2). One of the major V. cholerae virulence factors is cholera toxin, a secreted AB_5_ toxin that causes chloride ion imbalances in intestinal epithelial cells, resulting in the massive, watery, mucoid diarrhea that characterizes cholera (3). Cholera toxin is secreted to the extracellular milieu by the type II secretion system (T2SS), a widespread protein secretion pathway in Gram-negative pathogens (4–7).

The V. cholerae T2SS consists of 12 Eps (extracellular protein secretion) proteins, EpsC through EpsN, and PilD, and spans the entire cell envelope (8–11). The secretin EpsD forms the outer membrane channel of the T2SS and directly interacts with EpsC, which, together with EpsL, EpsM, EpsF, and EpsN, form the cytoplasmic membrane complex. The pseudopilus, composed of EpsG, EpsH, EpsI, EpsJ, and EpsK, extends from the cytoplasmic membrane complex and is actively involved in moving the type II secreted proteins from the periplasmic compartment through the secretin in the outer membrane in a process that requires energy generated by the ATPase EpsE (5, 12). In addition to cholera toxin, the T2SS in V. cholerae is responsible for the secretion of biofilm matrix proteins, chitin-binding and -degrading proteins, lipases, hemagglutinin/protease (HAP), a collagenase, and the serine proteases VesA, VesB, and VesC (7, 8, 13–18).

Proteins secreted by the T2SS are produced with N-terminal signal peptides that direct them to the periplasm, where they fold and connect with the T2SS for outer membrane translocation. In the absence of a functional T2SS, these proteins accumulate in the periplasmic compartment. Inactivation of the T2SS in V. cholerae also results in reduced growth rate in rich media, cell envelope perturbations, increased sensitivity to bile and polymyxin B, and induction of the stress response regulator RpoE (9, 19–21). Growth defects have also been reported for *pilD* mutants of V. cholerae, which lack the prepilin peptidase shared by the T2SS and one of the type IV pilus systems (11). Additional reports of analogous phenotypes among T2SS mutants have been observed in Vibrio vulnificus, *Vibrio* sp. strain 60, and Aeromonas hydrophila (22–25). Other studies have suggested that the V. cholerae T2SS genes are essential (26–29). For example, using a transposon-based approach to identify genes required for growth of the V. cholerae strain N16961, Judson and Mekalanos categorized *epsD* and *epsG* as essential (26). The genes *epsD* through *epsG*, *epsI*, *epsK*, and *epsL* and *pilD* were also identified as putatively essential genes by Cameron et al. and Kamp et al., as transposon insertions in these genes were not identified during genome-saturating transposon screens, presumably because bacteria containing transposon insertions in these genes could not be recovered (27, 29). However, in a study that categorized V. cholerae genes as essential, domain essential (containing both essential and nonessential coding regions), or sick, transposon insertions in some of the *eps* genes that had been reported as essential by others resulted in viable but sick mutants (28). Collectively, these studies suggest that secondary mutations arise to suppress a potentially lethal phenotype associated with loss of function of the T2SS. To test this hypothesis, we subjected six T2SS mutants to high-throughput genome sequencing and identified secondary mutations in all mutants. The finding that two of the mutants had acquired distinct mutations in the same gene, *vesC*, prompted us to further interrogate this gene in 92 additional *eps* mutants by PCR amplification and Sanger sequencing. This process identified another 19 *eps* mutants with unique *vesC* mutations, suggesting a selective pressure to lessen the stress and potential lethal phenotype induced by T2SS mutations in V. cholerae.

## RESULTS

### Inactivation of the type II secretion system in V. cholerae reduces growth rates.

**FIG 1:**
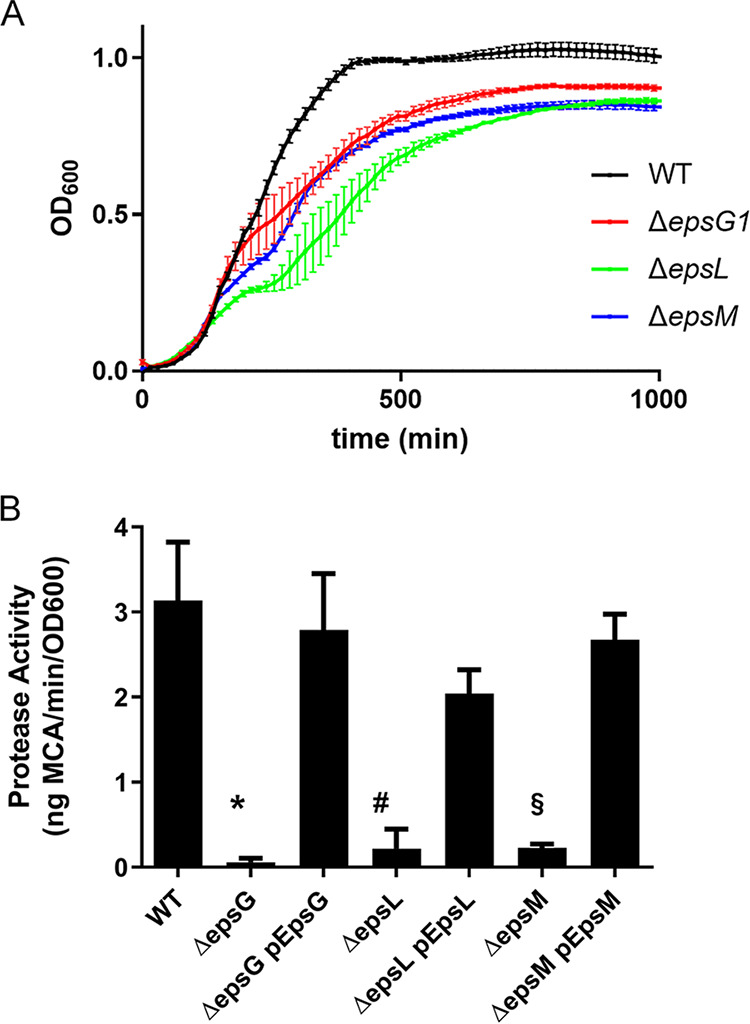
Vibrio cholerae T2SS mutants display reduced growth rates and lack extracellular serine protease activity. (A) Stationary-phase cultures of WT and mutant strains of V. cholerae were back-diluted to an *A*_600_ of 0.05 and inoculated into microtiter plates in duplicate. The *A*_600_ was measured using a Bioscreen growth curve analyzer every 15 min for 20 h. Experiments were performed in triplicate, and means ± SD are displayed. (B) Complementation of *eps* genes in V. cholerae T2SS mutants restores extracellular protease activity. Protease activity was measured in overnight culture supernatants using a fluorogenic peptide as described in Materials and Methods. Data shown are the means ± SD from at least three independent experiments. *, *P* < 0.001 versus WT and Δ*epsG1*pEpsG; #, *P* < 0.001 versus WT and Δ*epsL*pEpsL; §, *P* < 0.001 versus Δ*epsM*pEpsM (one-way analysis of variance [ANOVA] with Tukey’s multiple-comparison test).

We have previously reported that a V. cholerae mutant lacking all *eps* genes exhibits a growth rate reduction compared to the isogenic wild-type (WT) strain, suggesting that loss of the T2SS results in a slower growth phenotype (19). Strains containing inactivating mutations in single *eps* genes also grow slower. For example, the Δ*epsG1*, Δ*epsL*, and Δ*epsM* mutants that are lacking the genes encoding EpsG, EpsL, or EpsM, which directly interact within the T2SS complex (30–33), show reduced growth rates compared to T2SS-competent WT isolates (Fig. 1A). The colony size of the T2SS mutants is also consistently smaller than that of WT strains. As reported previously, inactivation of these *eps* genes abolishes secretion as measured here by the loss of extracellular serine protease activity (Fig. 1B) (9, 13, 19, 34, 35).

### Identification of secondary mutations in V. cholerae T2SS mutants.

We hypothesized that introducing *eps* mutations selects for secondary mutations that suppress a potentially lethal phenotype associated with loss of secretion by the T2SS. Thus, we sought to identify secondary mutations among V. cholerae T2SS mutants using high-throughput genome sequencing. We chose to focus on mutants with deletions of *epsG*, *epsL*, and *epsM*, as these genes have been reported to be essential by some and nonessential or generating a sick phenotype when mutated by us and others. Using Illumina Hi-Seq technology, we sequenced the genomes of the V. cholerae El Tor strain TRH7000, a *ctxAB*::Hg^R^ derivative of N16961, and the isogenic Δ*epsG1*, Δ*epsL*, and Δ*epsM* mutants. To identify genetic differences (including single-nucleotide polymorphisms [SNPs] and structural variants [SVs]) between the T2SS mutants and WT V. cholerae, we used reference-guided alignment using SeqMan software Lasergene (DNASTAR, Madison, WI) with the sequenced strain N16961 as a template. We then subtracted any SNPs or SVs found between TRH7000 and N16961. This list was then used to establish a list of SNPs or SVs unique to each of the sequenced T2SS mutants. Two secondary mutations were identified in the Δ*epsG1* and Δ*epsL* mutants, while five mutations were identified in the Δ*epsM* mutant, as tabulated in Tables 1, 2, and 3. Interestingly, the Δ*epsG1* and Δ*epsL* mutants have acquired distinct mutations in the *vesC* gene (VC1649), which encodes a T2SS-secreted protease (13, 36). The Δ*epsG1* mutant contains a 7-bp insertion (frameshift mutation) at position 1467 of *vesC*, resulting in a premature stop codon at amino acid (aa) 492 of this 548-aa protein (491fs). The Δ*epsL* mutant harbors a point mutation altering residue 279 from a glutamine to a proline (Q279P) in VesC. In both Δ*epsG1* and Δ*epsL* mutants, one additional gene contains a mutation besides *vesC*, and these are unique between the two strains. Specifically, the Δ*epsG1* mutant contains a mutation in *rfbV* (VC0259), a gene required for lipopolysaccharide biogenesis (37), and the Δ*epsL* strain contains a mutation in a putative secreted glycoside hydrolase gene, VCA0254. The Δ*epsM* mutant contains secondary mutations in five genes that encode proteins annotated as membrane proteins, a metabolic enzyme, and a ribosomal protein (Tables 1, 2, and 3). While the *rfbV* mutation in the Δ*epsG1* strain was confirmed by PCR amplification and Sanger sequencing of the genomic DNA that was subjected to whole-genome sequencing, it was absent in the DNA isolated from three new cultures of the original Δ*epsG1* freezer stock. That only 72% of the reads (Table 1) carried this mutation suggests that the mutation occurred during the growth of the culture used for whole-genome sequencing. The mutation in *vesC* was confirmed in the original freezer stock, as were the two mutations in the Δ*epsL* mutant. To further demonstrate that a variety of secondary mutations arise when *eps* genes are inactivated, we subjected three additional independently isolated mutants to whole-genome sequencing. These mutants included a second *epsG* mutant, here called Δ*epsG2*, and two mutants, called PU3 and PU5, with transposons at two different positions in *epsM* (8). These mutants also carry secondary mutations (Tables 1, 2, and 3), but they differ from those of the Δ*epsG1*, Δ*epsL*, and Δ*epsM* mutants.

**TABLE 1:** Overview of SNPs and structural variants in V. cholerae T2SS mutants

Gene ID	Description	Reads (%) that contain an SNP^*a*^					
		Δ*epsL*	Δ*epsG1*	Δ*epsG2*	Δ*epsM*	PU3	PU5
VC0259	RfbV		72				
VC0286	Gluconate permease				100		
VC0613	β-*N*-acetylhexosaminidase				100		
VC1133	HisD					100	
VC1649	VesC	100	SV				
VC1718	Hypothetical				100		
VC2252	BamA			100			
VCA0254	Hypothetical	98					
VC2701	DsbD					SV	
VC2506	HepA						SV
VC1915	RpsA				SV		
VC2323	Hypothetical				SV		

a SNP, single-nucleotide polymorphism; SV, structural variant.

**TABLE 2:** Identity and location of SNPs in V. cholerae *eps* mutants^*a*^

Mutant	Feature	Function	Impact	SNP (%)	DNA change	Amino acid change	Coverage depth
Δ*epsL*	VC1649	VesC	Nonsynonymous	100	c. 836A>C	p. Q279P	302
	VCA0254	Hypothetical	Frameshift	95	c. 1645delG		406
Δ*epsG1*	VC0259	Lipopolysaccharide biosynthesis protein RfbV	Frameshift	72	c. 594_595insA	p. K199fs	247
Δ*epsG2*	VC2252	Outer membrane protein assembly factor BamA	Nonsynonymous	100	c. 1502T>C	p. I501T	85
Δ*epsM*	VC0286	Gluconate permease	Nonsynonymous	100	c. 298A>T	p. I100F	139
	VC0613	β-*N*-acetylhexosaminidase	Nonsynonymous	100	c. 1012G>A	p. G338S	116
	VC1718	Hypothetical	Nonsynonymous	100	c. 568G>A	p. V190M	67
PU3	VC1133	HisD	Synonymous	100	c. 927A>G	p.(=)	212

a “c.” denotes nucleotide change at the indicated position; "p." denotes amino acid change at the indicated position.

**TABLE 3:** Unique structural variants in V. cholerae *eps* mutants

Mutant	Feature	Function	DNA change	Start location	Coverage depth
Δ*epsG1*	VC1649	VesC	7-nt insertion	1466	270
Δ*epsM*	VC1915	30S ribosomal protein S1	33-nt insertion	1576	49
	VC2323	Hypothetical protein	10-nt insertion	374	125
PU3	VC2701	DsbD	60-nt insertion	254	230
PU5	VC2506	ATP-dependent helicase HepA	124-nt insertion	1859	522

While the majority of secondary mutations in the mutants with deletions or insertions in *epsG*, *epsL*, or *epsM* may be directly or indirectly linked to the cell envelope, here we chose to follow up with the Δ*epsG1* and Δ*epsL* strains for further characterization, because they contained different mutations in the same gene, *vesC*, indicating one possible conserved mechanism for suppressor mutations to reverse a potential lethal phenotype of V. cholerae T2SS mutants.

### Secondary mutations in *vesC* abolish protease activity.

**FIG 2:**
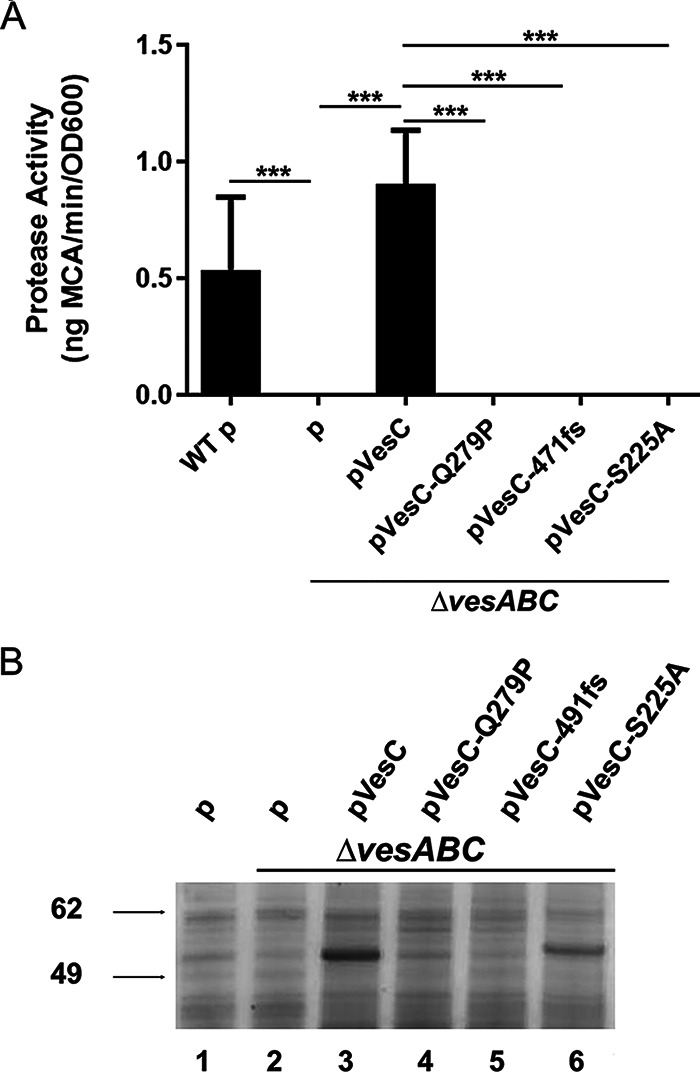
No serine protease activity is detected following expression of VesC-Q279P and VesC-491fs in V. cholerae with a functional T2SS. (A) Protease activity was measured in log-phase culture supernatants of V. cholerae strain N16961 (WT) containing an empty vector as well as the isogenic Δ*vesABC* strain containing empty vector or plasmids that code for WT VesC, VesC-Q279P, VesC-491fs, or VesC-S225A. Experiments were performed in triplicate or more, with means and SD shown. ***, *P* < 0.0001 (one-way ANOVA with Tukey’s multiple-comparison test). (B) Culture supernatants of overnight cultures of the strains analyzed in panel A were subjected to SDS-PAGE and silver staining.

To determine whether the secondary mutations in *vesC* harbored by Δ*epsG1* and Δ*epsL* mutants inactivate VesC, we cloned and overexpressed these mutant genes in a Δ*vesABC* mutant that lacks the genes for the VesA, VesB, and VesC proteases. The protease activity in the culture supernatants was determined and compared to that of supernatants from Δ*vesABC*-expressing genes for either WT VesC or an inactive variant, VesC-S225A, that has the catalytic serine in the active site replaced with alanine. Neither VesC-Q279P nor VesC-491fs restored extracellular protease activity in the Δ*vesABC* mutant, while protease activity was detected in the presence of WT VesC, indicating that these modifications in VesC abolish its activity (Fig. 2A). Culture supernatants of the same strains were also analyzed by SDS-PAGE and silver staining (Fig. 2B). While overexpression of WT VesC and the catalytically inactive VesC-S225A resulted in visible proteins of approximately 55 kDa (lanes 3 and 6), neither VesC-Q279P nor VesC-491fs variants were discernible above background, suggesting that they are unstable and/or not secreted (lanes 4 and 5).

### Additional mutations in *vesC*.

The possibility that other V. cholerae T2SS mutants also harbor mutations in the *vesC* gene was investigated next. Genomic DNA from 92 additional mutants with *eps* gene deletions/modifications and growth defects to various degrees were isolated, and the *vesC* gene from each mutant was amplified by PCR and subjected to Sanger sequencing. We identified 19 additional unique mutations in *vesC* in mutants with deletions of *epsC*, *epsD*, *epsE*, *epsF*, *epsG*, and *epsL* (Tables 4 and 5). These included frameshift mutations due to insertions or deletions, sequence duplications, and a variety of single-nucleotide changes resulting in amino acid substitutions in the VesC protein.

**TABLE 4:** SNPs in *vesC* in additional V. cholerae *eps* mutants

Mutant^*a*^	DNA change	Amino acid change	Impact
TRH Δ*epsC6*	c. 1292C>T	p. P431L	Nonsynonymous
TRH Δ*epsC10*	c. 738delG	p. 246fs	Frameshift
TRH *gfp-epsM* Δ*epsC5*	c. 1067_1068insT; 1073_1074insTT	p. K357_L358insL	Frameshift; insertion
TRH *gfp-epsM* Δ*epsC6*	c. 1304T>C	p. L435P	Nonsynonymous
TRH Δ*epsD8*	c. 1282_1283insT	p. 427fs	Frameshift
TRH *gfp-epsM* Δ*epsD7*	c. 187A>C	p. S63R	Nonsynonymous
TRH *gfp-epsM* Δ*epsD8*	c. 829T>C	p. Y279H	Nonsynonymous
TRH Δ*epsE3*	c. 187A>C	p. S63R	Nonsynonymous
C7606 Δ*epsG*	c. 549_550insC	p. 185fs	Frameshift
TRH *gfp-epsM* Δ*epsL1*	c. 488G>A	p. R163H	Nonsynonymous
TRH *gfp-epsC* Δ*epsL1*	c. 476G>T	p. G159V	Nonsynonymous
TRH *gfp-epsC* Δ*epsL3*	c. 738delG	p. 246fs	Frameshift

a Most *eps* mutations were constructed in TRH7000 (abbreviated to TRH) or in TRH7000 expressing chromosomal fusions of *gfp* with either *epsC* or *epsM*.

**TABLE 5:** Unique structural variants identified in *vesC* of additional *eps* mutants

Strain	DNA change	Start location	Notes
TRH *gfp-epsM* Δ*epsC1*	15-nt deletion	193	5-aa deletion of residues Y64–R68
TRH Δ*epsD6*	10-nt deletion	1489	Frameshift
TRH *gfp-epsC* Δ*epsD14*	15-nt duplication	201	186_201dupAGTTATCTTGGTGGT, duplication of 5 amino acids, S63–G67
TRH Δ*epsE6*	4-nt insertion	933	Frameshift
rA1552 Δ*epsF*	11-nt insertion	1385	Frameshift
TRH Δ*epsL3*	161-nt deletion	1207	Frameshift
TRH *gfp-epsC* Δ*epsL2*	14-nt deletion	187	

### Structure of VesC.

To explain the effect of the VesC alterations, we obtained the three-dimensional structure of VesC. Optimized crystals that diffracted to 2.2-Å resolution were produced from the construct pro-VesC containing residues 23 to 522, which includes the propeptide but not the N-terminal signal peptide or the C-terminal GlyGly-CTERM motif (see Fig. S1 in the supplemental material). The structure of VesC was determined by the molecular replacement method assisted by Rosetta homology modeling with electron density using VesB protease (PDB entry 4LK4) and carbohydrate binding module (CBM) domains (PDB entries 1UXX and 2C9A) as search models (38). The final structure was refined to an R-factor of 0.201 and an R-free of 0.234 with good geometry (Table 6).

**TABLE 6:** Data collection and refinement statistics

Parameter	Value
Data collection	
Beamline	SSRL14-1
Wavelength (Å)	0.97939
Space group	*P*2_1_2_1_2_1_
Unit cell-parameters (Å, °)	*a *=* *41.65, *b *=* *83.40, *c *=* *123.11, α = β = γ = 90
Resolution range (Å)	39.5–2.20 (2.26–2.20)^*a*^
No. of total reflections	163,023
No. of unique reflections	22,516
Completeness (%)	100.0 (100.0)
Mean *I*/σ(*I*)	15.6 (2.27)
*R*_merge_	0.096 (0.969)
Multiplicity	7.2 (7.0)
CC_1/2_^*b*^	99.8 (69.7)
Refinement statistics	
Resolution range (Å)	39.5–2.20
No. of unique reflections	22,515
No. of reflections for *R*_free_	113
*R*_work_/*R*_free_	0.201/0.234
RMSD, bond lengths (Å)	0.020
RMSD, bond angles (°)	1.704
*B* factor (Å^2^)	
Wilson *B*	43.1
Overall	37.6
Protein	38.2
Solvent	33.5
No. of non-H atoms	
Protein	3,527
Water	124
Ramachandran statistics^*c*^ (%)	
Most favored	98.23
Additionally allowed	1.33
Disallowed	0.44

a Values in parentheses are for the highest-resolution shell.

b CC_1/2_, correlation coefficient as defined in Karplus and Diederichs (69) and calculated by *XSCALE* (53).

c Calculated using the MolProbity server (70).

10.1128/mSphere.01125-20.1 FIG S1 Structure-based sequence alignment of VesC and VesB. The protease domains are shaded in gold, the Ig fold domains are shaded in green, and the CBM domain of VesC is shaded in blue. Secondary structure elements corresponding to VesC (this work; PDB entry 6BQM) and VesB structures (S. Gadwal, K. V. Korotkov, J. R. Delarosa, W. G. J. Hol, and M. Sandkvist, J Biol Chem 289:8288–8298, 2014; PDB entry 4LK4) are displayed above and below the alignment, respectively. The catalytic triad residues are highlighted in blue. The signal peptides are shown in gray boxes. The black vertical arrow indicates the position of the propeptide processing site. The conserved C-terminal region that contains the Gly-Gly-CTERM motif is shown in a black box. Blue circles highlight the residues of the Ig fold domain of VesC that are in contact with the protease domain. The mutations identified in VesC are indicated by orange arrows. Download FIG S1, JPG file, 0.4 MB. Copyright © 2020 Rule et al. 2020 Rule et al. This content is distributed under the terms of the Creative Commons Attribution 4.0 International license.

**FIG 3:**
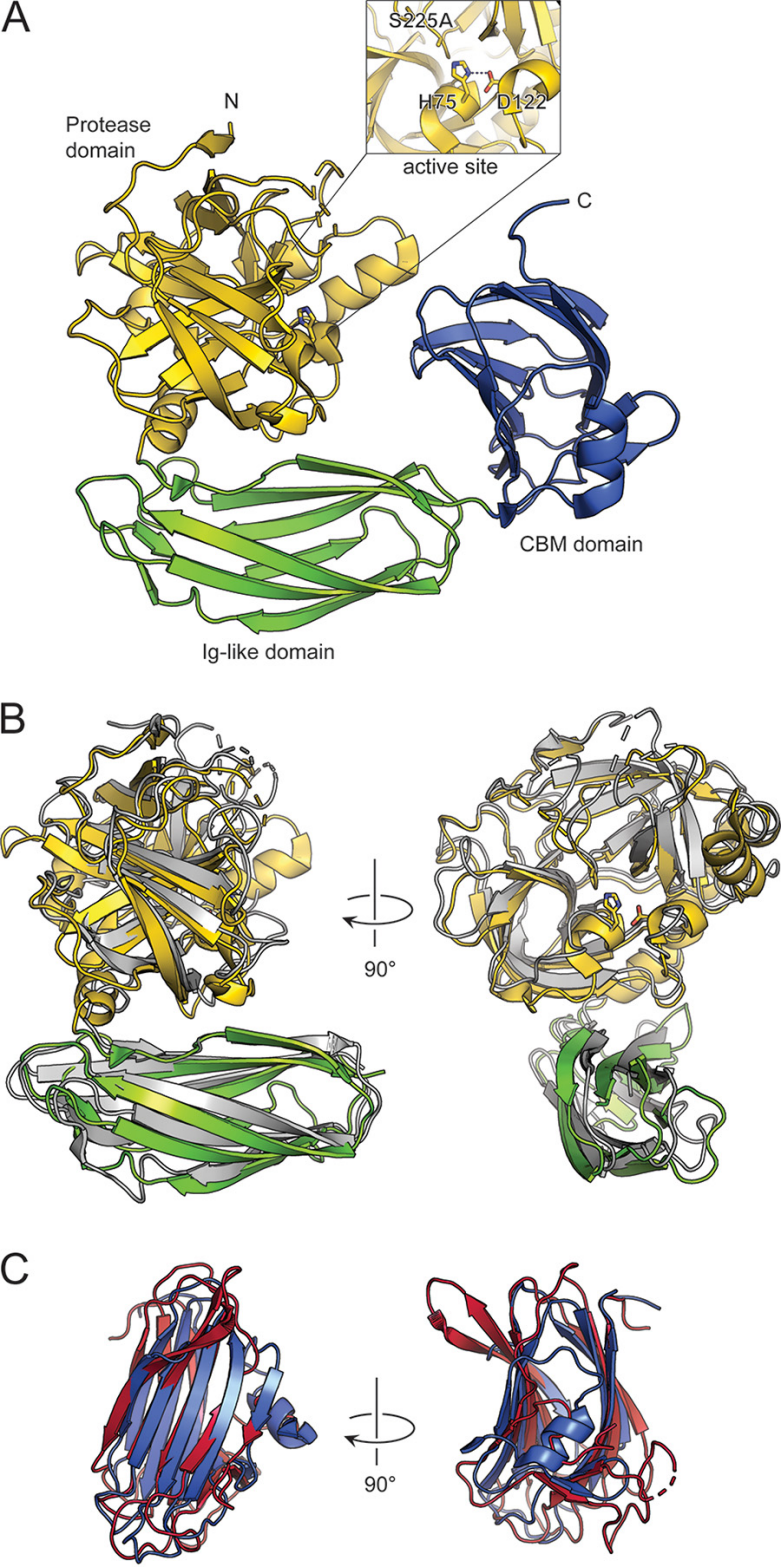
Crystal structure of V. cholerae VesC. (A) Structure of VesC is shown in ribbon representation with the protease domain in gold, the Ig-like domain in green, and the CBM domain in blue. The inset shows the active-site residues in stick representation. (B) A structural superposition of VesC and VesB. The protease domain of VesC is in gold, the Ig-like domain of VesC is in green, and VesB is in gray. VesB lacks a CBM domain; hence, no superposition of CBM domains is possible. (C) A structural superposition of the CBM domain of VesC (blue) and Meprin A subunit beta from Homo sapiens (PDB entry 4GWM) (red).

The overall structure of VesC revealed that it consists of three domains: an N-terminal domain with a protease fold, the middle domain with an Ig-like domain, and the C-terminal domain with a β-sandwich fold (Fig. 3A). Well-defined electron density maps allowed the fitting of all three domains, the protease fold (35 to 272), the Ig-like fold (273 to 380), and the C-terminal domain (381 to 511), with the exception of the N-terminal residues (23–34), including the propeptide and C-terminal tail residues (512 to 522), due to their flexibility in the crystal structure. Three disulfide bonds are found in the protease domain (C60–C76 and C190–C212) and Ig-like domain (C330–C340), which are also conserved in VesB (PDB entry 4LK4).

The structural comparison of the protease domain of VesC (VesC^PD^) with typical trypsins showed a high degree of structural similarity, with the best hit found in VesB^PD^. The structure of VesC^PD^ can be superimposed onto the VesB^PD^ structure (PDB entry 4LK4) with a root mean square deviation (RMSD) of 1.8 Å with 42% sequence identity for 205 residues (Fig. 3B). Consistent with the structures of VesB^PD^ and other trypsinogens, VesC^PD^ displays a similar positioning of its catalytic residues, and the three disordered loop regions (residues 164 to 172, 216 to 220, and 247 to 249) in the position of the active site are also observed in the crystal structure of VesC^PD^. In contrast, two loop regions, residues 115 to 121 and 194 to 208, in VesC^PD^ adopt substantially different conformations, which extend upwards from the globular body of the protease domain.

The Ig domain of VesC (VesC^Ig^) is 22% identical and 55% similar in amino acid sequence to VesB^Ig^. In spite of this moderate sequence homology, the structures of VesC^Ig^ and VesB^Ig^ are very similar (Fig. S1 and S3B). Additionally, the two-domain structures of VesC^PD+Ig^ and VesB^PD+Ig^ are relatively similar, with an RMSD of 2.3 Å for 301 equivalent Cα atoms. In the crystal structure, the interface between VesC^PD^ and VesC^Ig^ buries a 1,568-Å^2^ solvent-accessible surface, which is ∼28% larger than the interface between the homologous domains in VesB.

The structure of the third C-terminal domain of VesC, which is missing in VesB, revealed a concave β-sandwich domain (Fig. 3A). A homology structure search using the DALI server showed that the human proteins meprin A subunit beta, receptor-type tyrosine protein phosphatase MU, reelin, and the CBM family 29 of NCP-1 from *Piromyces equi* are the closest structural homologs of VesC^CBM^ (Table 7). Interestingly, the closest structural homologs found by secondary-structure matching (SSM) PDBeFold include distant homologs with CBMs (PDB entries 1OH3, 2ZEX, 2ZEZ, and 2XOM). Comparing VesC^CBM^ with CBM family 29 (PDB entry 1OH3) revealed that the two structures share 73% of their secondary-structure elements. Although VesC^CBM^ has a low level of sequence similarity to the previously determined crystal structures of CBMs, the superposition shows the conserved core β-sandwich composed of 10 β strands (Fig. 3C). These CBM families all adopt the β-sandwich scaffold, displaying a curved platform with diverse carbohydrate binding modes in the variable loops and the concave face and residues with aromatic side chains (39). The high degree of structural similarity between VesC^CBM^ and CBM family proteins suggests that VesC binds to carbohydrates and/or target glycosylated substrates.

**TABLE 7:** Structural homologs of VesC^CBM^^*a*^

Chain	Z	RMSD	Lali	Nres	% identity	Description
4gwm-A	12.0	2.6	117	561	15	Human meprin A subunit beta
2v5y-A	11.3	2.4	117	564	22	Human receptor-type tyrosine-protein phosphatase Mu
2ddu-A	10.2	2.2	107	301	10	Human reelin
1oh3-A	10.1	2.5	106	141	12	CBM29 domain 2 of NCP-1, a component of the *P. equi* cellulase/hemicellulase complex

a Z, Z-score; Lali, length of the alignment; Nres, number of residues.

### Structural analysis of VesC substitutions.

**FIG 4:**
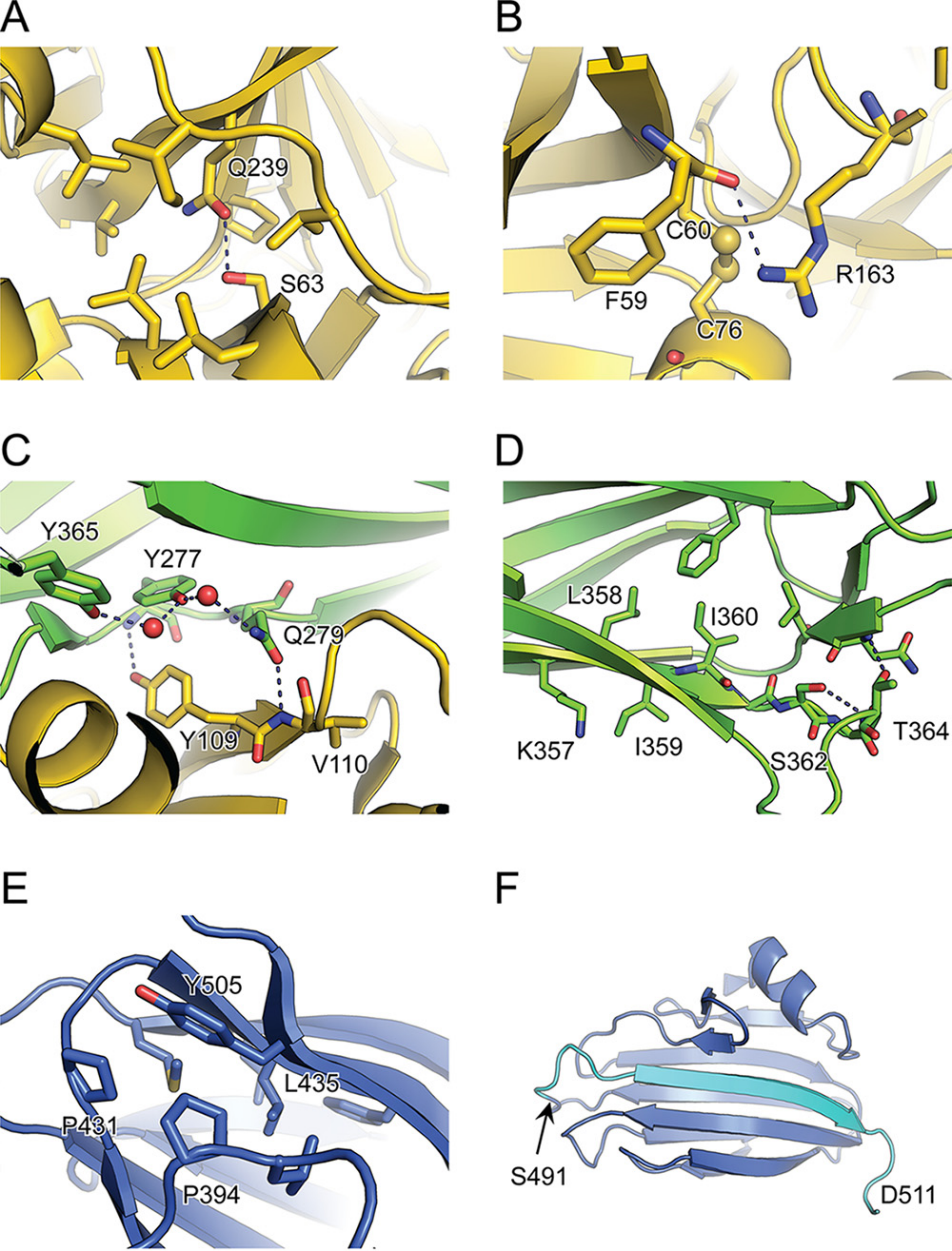
Structural environment of mutated residues in VesC. The substituted and neighboring residues are shown in stick representation. The colors correspond to those of Fig. 3. The local environment is shown for S63R (A), G159V (B), Y277H and Q279P (C), K357insL (D), and P431L and L435P (E). (F) The CBM domain of VesC in ribbon representation. The 491-frameshift alteration indicated by an arrow results in truncation of the last β-strand (cyan) that occupies a central position in one of the β-sheets of the CBM domain.

The VesC structure offers the opportunity to explain the effects of the mutations, which were scattered throughout *vesC*, on the folding, stability, and/or function of this T2SS substrate. The S63R substitution identified in two independent *eps* mutants is located on the N-terminal lobe of VesC^PD^. The hydroxyl of S63 forms a buried hydrogen bond with Q239 (Fig. 4A). To evaluate the possibility of acceptable substitutions at this position, we performed sequence tolerance analysis using Rosetta backrub (40). Serine was the preferable residue tolerated at this position (Fig. S2A). The loss of the buried hydrogen bond and the bulky arginine sidechain resulting from the S63R substitution likely lead to destabilization of VesC^PD^. Similarly, duplication of residues 63 to 67 and deletion of 64 to 68 in this region could result in misfolding of VesC^PD^. The G159V substitution is located in the C-terminal lobe of VesC^PD^. The backbone conformation of G159, φ 157.9° and ψ 177.9°, is favorable for glycine but not other residues. The sequence tolerance analysis showed that only glycine is acceptable at this position (Fig. S2B). The R163H variant is located downstream on the same loop as G159V. The guanidinium group of R163 makes a hydrogen bond with the carbonyl of F59 and is in van der Waals contact with the disulfide C60–C76 (Fig. 4B). The arginine residue is strongly preferred at this position based on sequence tolerance analysis (Fig. S2C).

10.1128/mSphere.01125-20.2 FIG S2 Sequence tolerance analysis of mutated positions in VesC. The distribution of tolerated substitutions at indicated positions as calculated by the Rosetta Backrub server (C. A. Smith and T. Kortemme, PLoS One 6, e20451, 2011) are shown as box and whisker plots. Download FIG S2, JPG file, 0.3 MB. Copyright © 2020 Rule et al. 2020 Rule et al. This content is distributed under the terms of the Creative Commons Attribution 4.0 International license.

10.1128/mSphere.01125-20.3 FIG S3 Comparison of domain interface interaction in VesC and VesB. VesC and VesB are shown in ribbon representation with the protease domain in yellow and the Ig-like fold in green. The CBM of VesC is shown in blue. The identical interdomain interface residues Q279 (VesC) and Q274 (VesB) are shown as ball representations. Download FIG S3, JPG file, 0.3 MB. Copyright © 2020 Rule et al. 2020 Rule et al. This content is distributed under the terms of the Creative Commons Attribution 4.0 International license.

The substitutions Y277H and Q279P are located in the VesC^Ig^ domain, with the Q279 side chain facing the VesC^PD^ domain (Fig. 4C). The side chain of Y277 is surrounded by hydrophobic residues, while the hydroxyl moiety makes hydrogen bonds with water molecules bridging Q279 and Y365 (Fig. 4C). The sequence tolerance analysis indicated that tyrosine and phenylalanine residues are preferred at this position (Fig. S2D). Therefore, the introduction of the polar histidine side chain could result in misfolding of the VesC^Ig^ domain. The Q279 residue is present in the middle of the interface between VesC^Ig^ and VesC^PD^ (Fig. 4C and Fig. S3). Ninety-one percent (71 Å^2^ out of 78 Å^2^) of accessible surface area of Q279 is buried in the VesC^Ig^-VesC^PD^ interface that includes a hydrogen bond with the main chain of V110 (Fig. 4C). The sequence tolerance analysis showed that glutamic acid and glutamine residues are preferred at position 279 (Fig. S2E). Since the side chain of proline is markedly different from that of glutamine, the Q279P alteration likely modifies the domain interaction and prevents proper folding of VesC. Structural analysis indicates that the analogous glutamine residue in VesB similarly forms part of an interface with residues in the protease domain (Fig. S3). Previous attempts to produce extracellular VesB without its Ig-fold domain in V. cholerae have been unsuccessful, supporting the suggestion that this domain plays an important role in protein stabilization and/or secretion of VesB and possibly also VesC (41).

The probable deleterious effect of leucine residue insertion after K357 in the VesC^Ig^ domain could be explained by an effect on adjacent positions, because K357 is followed by L358 (Fig. 4D). Therefore, this would effectively lead to an I359L substitution and the extension of the downstream loop by one residue. Both of these changes would likely decrease the stability of VesC^Ig^, because a small hydrophobic residue, valine or isoleucine, is preferred at position 359 (Fig. S2F), and all residues of the downstream loop are engaged in specific contacts (Fig. 4D).

Substitutions P431L and L435P are located in the VesC^CBM^ domain. Residue P431 is engaged in van der Waals contacts with P394 and Y505 (Fig. 4E), and sequence tolerance analysis indicated that a small residue is preferred at this position (Fig. S2G). Residue L435 is oriented toward the hydrophobic core of VesC^CBM^, and substitution with a smaller proline moiety may destabilize this domain. Additionally, φ/ψ angles of L435 would be less preferable for a P residue. Sequence tolerance analysis showed a strong preference for leucine at this position (Fig. S2H). Furthermore, the S491 frameshift in VesC resulting in a premature stop at residue 492 is positioned in the middle of the β-sandwich fold of the VesC^CBM^ structure, removing the C-terminal 56 residues, including a structurally important β-strand and the GlyGly-CTERM domain (Fig. 4F). Finally, a large D403-456 deletion would effectively eliminate VesC^CBM^. In summary, all observed alterations in VesC would negatively impact its stability and/or folding, preventing the production of an active VesC enzyme.

### Overexpression of WT VesC affects growth of the *ΔepsG1* mutant.

**FIG 5:**
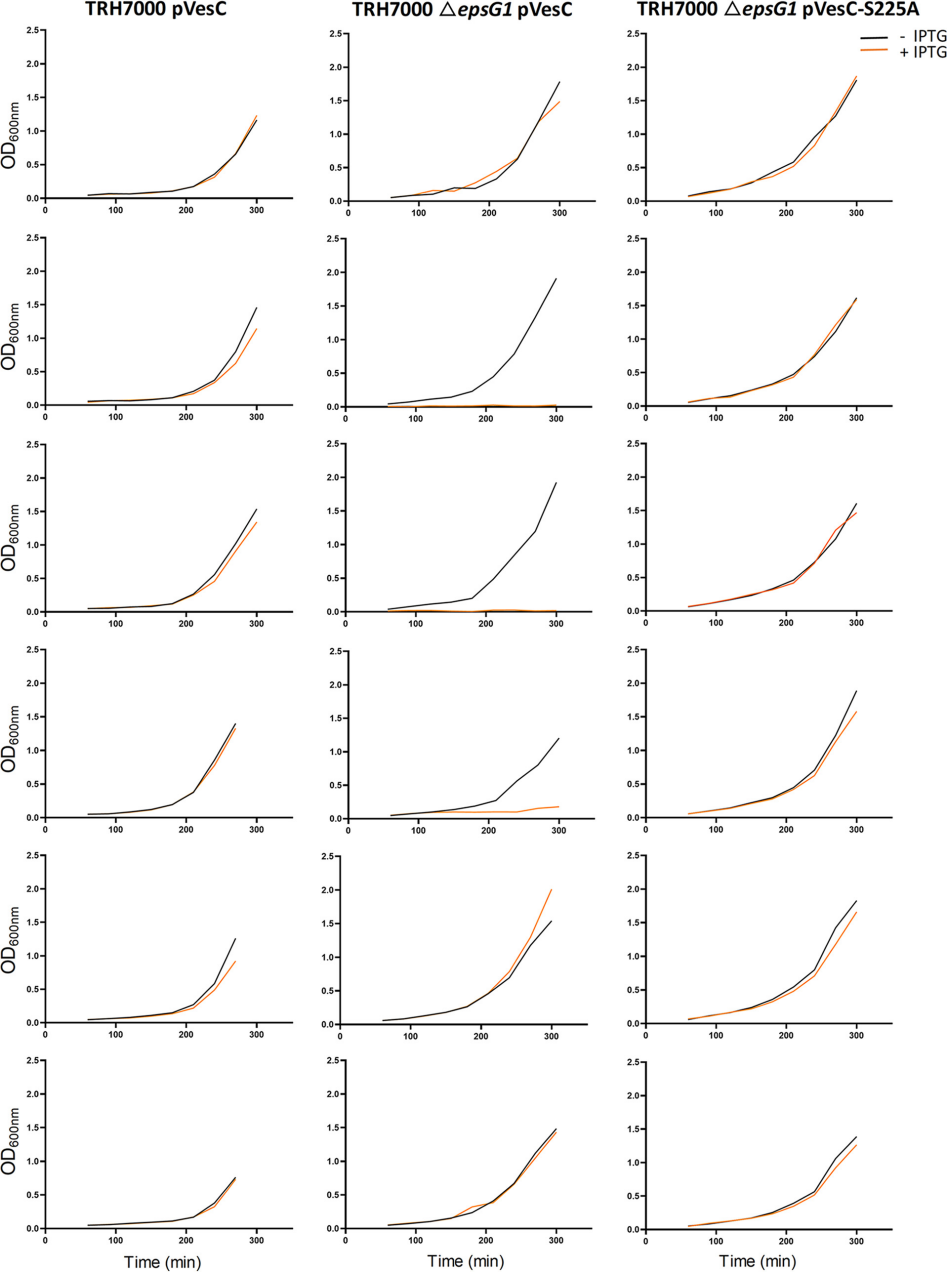
Overexpression of VesC interferes with growth of the T2SS Δ*epsG1* mutant. WT V. cholerae TRH7000 (left column) and the Δ*epsG1* mutant (middle column) containing a plasmid encoding WT *vesC* were grown in the absence (black line) or presence of IPTG (orange line) to induce the production of VesC. Optical density at 600 nm (OD_600nm_) of the cultures was monitored over time (shown in minutes). The Δ*epsG1* mutant producing the catalytically inactive VesC-S225A variant (right column) was similarly grown without and with IPTG.

To determine whether expression of *vesC* has a negative impact on cells that are deficient in extracellular secretion, we overexpressed WT *vesC* in the Δ*epsG1* mutant. Following growth overnight, cultures were diluted in fresh media, split in half, and grown in the presence or absence of 50 μM isopropyl-d-thiogalactopyranoside (IPTG). All cultures grew without IPTG, whereas three of six cultures did not grow in the presence of IPTG (Fig. 5). In control experiments, all WT V. cholerae TRH7000 cultures grew as well with as without IPTG. Finally, no growth inhibition was observed when the proteolytically deficient VesC-S225A was overexpressed in the Δ*epsG1* mutant, suggesting that VesC activity is the cause of toxicity when its secretion is impeded.

## DISCUSSION

This study was initiated to determine why T2SS mutants of V. cholerae are viable even though several of the genes encoding the T2SS have been deemed essential (26–29). We hypothesized that V. cholerae T2SS mutants acquire secondary mutations that suppress a potential lethal phenotype. Indeed, we confirmed the presence of secondary mutations in each of the six sequenced V. cholerae T2SS mutants. Two of the six *eps* mutants acquired distinct mutations in the same gene, VC1649, which encodes the T2SS-secreted serine protease VesC. This and the finding that additional unique *vesC* mutations are present in 19 out of 92 additional T2SS mutants suggests that one method by which *eps* mutations can be generated in V. cholerae is to inactivate one of its secreted substrates, which may otherwise cause damage when accumulating in the periplasm. Mutations in the *vesC* gene are not sufficient, however, to restore growth to WT levels. This was exemplified by the finding that cumulative mutations can appear during further growth of mutants such as the Δ*epsG1* mutant, which picked up a mutation in a second gene, *rfbV*, when cultured for the isolation of genomic DNA used for whole-genome sequencing (Table 1). We identified *vesC* mutations in mutants that are functionally lacking six of the 13 T2SS components (EpsC through EpsG and EpsL). The reason we have not detected *vesC* mutations in strains deficient in EpsH through EpsK, EpsM, and PilD may not be related to their specific function in the T2SS, as they are also required for secretion, and it is likely due to the limited number of mutants that we have generated. Although alterations were identified in all three domains of VesC, a hot spot of changes in the protease domain was observed, with one identical substitution of residue 63 in two independently isolated mutants, a deletion of residues 64 to 68, and a duplication of 63 to 67 (Tables 4 and 5). Through careful analysis of the VesC structure, we determined that all *vesC* mutations presented in this study would very likely eliminate the formation of an active enzyme.

**FIG 6:**
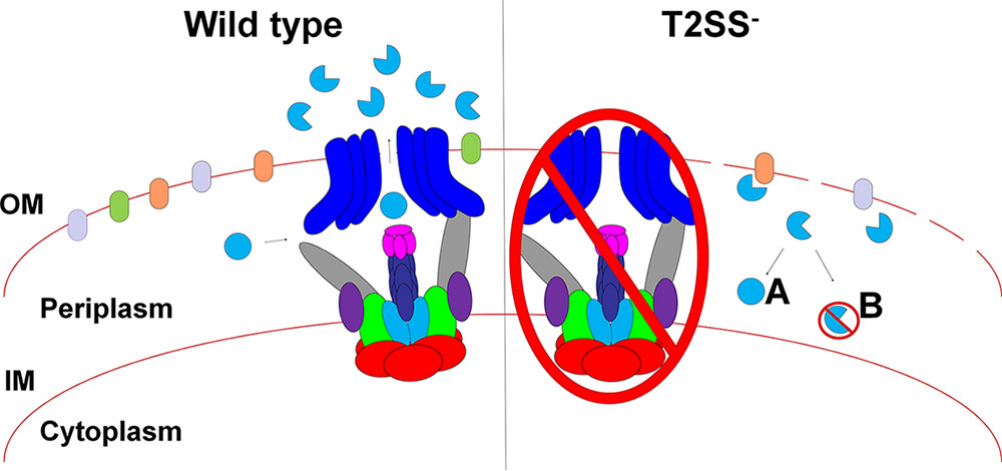
Working model of the mechanism by which secondary mutations in *vesC* may suppress a potential lethal phenotype of T2SS mutants. (Left) Wild-type V. cholerae transports VesC across the outer membrane via the T2SS. Upon inactivation of the T2SS, VesC secretion is blocked and the protease accumulates in the periplasm, where it may be a contributing factor to cell envelope damage through nonspecific proteolysis and a possible lethal phenotype (right). During the process of genetic inactivation of the T2SS, we may select for mutations that inactivate VesC (A) and/or target VesC for degradation (B) to prevent proteolysis of essential components and/or avert irreparable cell envelope damage in the absence of a functional T2SS.

Over 20 proteins are secreted by the T2SS, yet we identified secondary mutations in the same gene, *vesC*, in 21 different *eps* mutants. Perhaps whole-genome sequencing of additional *eps* mutants besides the six mutants sequenced here will reveal secondary mutations in genes encoding other T2SS substrates, but the observation of multiple distinct mutations in the *vesC* gene is indicative of a conserved mechanism for putative *eps* mutant suppression. The finding that three out of six cultures of the Δ*epsG1* mutant did not grow when WT *vesC* expression was induced from a plasmid with IPTG, while overexpression of *vesC* in the WT strain did not diminish growth, suggests that periplasmic accumulation of VesC is harmful and that VesC-inactivating alterations in T2SS mutants relieve this toxicity (Fig. 6). No growth inhibition was observed when the proteolytically deficient VesC-S225A was overexpressed in the Δ*epsG1* mutant, suggesting that VesC activity and not amount is the cause of toxicity. The reason why WT *vesC* expression did not interfere with growth of all Δ*epsG1* mutant cultures may be due to the appearance of additional suppressor mutations during culturing of the mutant containing the pVesC plasmid. Although we have only completed the sequencing of six T2SS secretion mutant genomes, we do not believe that the V. cholerae T2SS mutants carry inactivating mutations in the VesC-homolog VesB, as complementation of defective *eps* genes generally restores serine protease activity to 80 to 100% of WT activity (as exemplified in Fig. 1B), and VesB contributes to approximately 80% of secreted serine protease activity toward the fluorogenic Gln-Ala-Arg peptide (13). Thus, VesB and VesC both are capable of cleaving this short peptide; however, they likely differ in protein substrate specificity, as their protease domains are only 42% identical and VesC contains an additional domain. An alternative explanation for why VesC and not VesB (or VesA) is toxic when not secreted may be due to its higher level of production, as we have previously found that 3.5 times more VesC than VesB and VesA is detected in the culture supernatant of WT V. cholerae (13).

Mutations in genes that encode proteins essential for cell viability may have been selected to prevent their possible proteolysis by accumulating VesC in the periplasm of T2SS mutants. For example, BamA, an essential component of the beta-barrel complex required for outer membrane protein biogenesis, has an I501T substitution in the Δ*epsG2* mutant. Sequence-based structural modeling of V. cholerae BamA on E. coli BamA suggests that this residue is located in one of the cell surface-exposed loops. PU5, with a transposon in *epsM*, carries a 124-nt frameshift insertion in VC2506, which encodes the RNA polymerase-associated protein HepA. It is an ATP-dependent helicase that contributes to recycling of RNA polymerase during stress in E. coli (42). The effect of this mutation is not understood, but it may result in modification of gene expression to accommodate cellular stress in PU5. The other transposon mutant, PU3, has a 60-nt insertion in VC2701, which encodes a homolog of the E. coli DsbD. This results in a 20-amino-acid insertion in the first periplasmic domain of this inner membrane protein that contributes electrons to disulfide isomerases such as DsbC (43) and, thus, has an indirect function in disulfide isomerization of proteins in the periplasm. The second mutation is a synonymous mutation (A to G) at codon 927 that encodes a Ser in HisD, predicted to be involved in histidine metabolism. The frequency of the two Ser codons TCA and TCG in E. coli is the same, so it is not understood if the mutation has any effect on the synthesis of HisD in PU5, unless codon usage is different in V. cholerae. With five mutations, the Δ*epsM* mutant has the most secondary mutations of the sequenced strains. VC0613 encodes a predicted beta-N-acetylhexosaminidase and is part of a ChiS-regulated chitin-induced catabolic operon that contributes to amino sugar and nucleotide sugar metabolism in V. cholerae (44). It is possible that when V. cholerae is unable to secrete its two extracellular chitinases, there is no need for intracellular enzymes that participate in the downstream processing of chitin. In addition, the Δ*epsM* mutant has secondary mutations in three genes coding for inner membrane proteins. VC1718 encodes an ElyC homolog that is predicted to span the membrane twice. In E. coli, this protein is involved in cell wall precursor metabolism. The absence of ElyC results in defects in peptidoglycan synthesis at low temperatures (45). Residue Val190, which is replaced with a Met residue in the Δ*epsM* mutant, is predicted to be localized in the periplasmic domain. Another mutation is in VC0286, which encodes a gluconate permease. This protein is predicted to span the inner membrane 11 to 12 times and contributes to carbon metabolism (46). The mutation results in a substitution of Ile for Phe at position 100. The third mutation affecting an inner membrane protein is found in VC2323, a membrane protein that spans the membrane eight times and is homologous to the E. coli tellurite resistance protein. In V. cholerae, this protein may serve as an efflux pump that contributes to chloramphenicol resistance and intestinal colonization in infant mice (47). Finally, the fifth mutation is a 33-nucleotide insertion in VC1915, which encodes the 30S ribosomal protein S1. The 11-amino-acid insertion likely does not inactivate this protein, as it is essential for protein synthesis; however, it may alter the rate of protein synthesis to accommodate the stress associated with inactivation of the T2SS. The 73 *eps* mutants that lack mutations in *vesC* and have not been subjected to whole-genome sequencing may have similar mutations in genes encoding cell envelope components that are sensitive to periplasmic VesC. Other possibilities include mutations in genes that code for factors controlling expression, folding, and/or activation of VesC.

Finally, other genes with suppressor mutations may encode yet-to-be-identified T2SS substrates that could cause damage to components of the cell envelope, including the inner and outer membranes and peptidoglycan, when accumulating in the periplasm of T2SS mutants. For example, a mutation was detected in VCA0254 in addition to the *vesC* mutation in the Δ*epsL* mutant. This gene encodes a protein that is 21% identical to and can be modeled on 3,6-anhydro-d-galactosidase, an exolytic enzyme produced by a marine G-negative species, Zobellia galactanivorans, that targets carrageenan oligosaccharides (PDB entry 5OPQ [48]). Based on the extracellular location of the *Z. galactanivorans* enzyme and that both proteins are expressed with N-terminal signal peptides, we speculate that VCA0254 encodes an extracellular protein possibly secreted by the T2SS in V. cholerae. However, cloning and overexpression of VCA0254 in WT V. cholerae did not result in a detectable protein in the culture supernatant when analyzed by SDS-PAGE and silver staining (not shown).

Although many bacteria use the T2SS to support secretion of proteases, VesC is one of three unique trypsin-like serine proteases found in V. cholerae, other *Vibrio* species, and related marine species, including Aeromonas hydrophila, and may have a nonspecific and perhaps toxic activity inside the cell compared to other T2SS-secreted proteases. Therefore, we speculate that perhaps the T2SS genes are not essential *per se* but rather that the phenotype observed with T2SS mutants of V. cholerae, V. vulnificus, *Vibrio* sp. strain 60, and *A. hydrophila* is due to damage caused by particular T2SS substrates when they accumulate in the wrong location. Additional investigation into the relationship between organisms exhibiting T2SS inactivation-associated envelope defects and their corresponding suites of secreted substrates may reveal a conserved mechanism for suppression of T2SS-associated phenotypes in these organisms.

## MATERIALS AND METHODS

### Bacterial strains and growth conditions.

Vibrio cholerae N16961 (El Tor), TRH7000 (*thy* Hg^r^ [*ctxA-ctxB*]), and mutants thereof were grown at 37°C in LB broth, which was supplemented with 100 mg/ml thymine. Plasmid-containing strains were grown in the presence of 200 μg/ml carbenicillin, and gene expression was induced with 10 μM isopropyl-d-thiogalactopyranoside (IPTG) for *epsG*, *epsL*, and *epsM* or 50 μM IPTG for *vesC* expression.

### Cloning.

With the exception of the Δ*epsM* mutant, the mutants used in this study for the whole-genome sequencing were constructed previously (8, 19, 49). The Δ*epsG2* mutant was constructed in the same way as the Δ*epsG1* mutant (49). Mutants that were used for PCR amplification and sequencing of the *vesC* gene were also made previously (8, 17, 20, 30, 49, 50).

The Δ*epsM* mutant was constructed by amplifying regions upstream and downstream of the *epsM* gene and introducing an internal kanamycin resistance cassette from pKD4 using the following primers: 5′ CAAGTCTTCTTGGCTGCGGT 3′ (forward [Fwd] primer for upstream fragment), 5′ CGAAGCAGCTCCAGCCTACACTTCTCCTTACTTGGGCTTCACC 3′ (reverse [Rev] primer for upstream fragment), 5′ CTAAGGAGGATATTCATATGGCGTGGAGGCTGATATGA 3′ (forward primer for downstream fragment), and 5′ CCGACACGACAGTACCAAGCTGC 3′ (reverse primer for downstream fragment). PCR products from the upstream and downstream regions were used as a template for another PCR using the first and last primers, which was used for chromosomal replacement as described previously (51).

Plasmids containing either WT or T2SS mutant variants of genes identified from whole-genome sequencing were constructed by amplifying the gene of interest from TRH7000 chromosomes and cloning into pMMB67EH. The primers used to amplify *vesC* (VC1649) are 5′ GAGGAGCTCTGGGAGTTATCAGAGGTATC 3′ (Fwd) and 5′ GAGGCATGCTGGCTATCGATAGATCAGAC 3′ (Rev). pMMB-VesC S225A was constructed from pMMB-VesC WT using PCR mutagenesis, with overlapping primers containing the point mutation 5′ CGCTTGTTCTGGTGACGCCGGTGGCCCTATCTTTTTTG 3′ (Fwd) and 5′ CAAAAAAGATAGGGCCACCGGCGTCACCAGAACAAGCG 3′ (Rev). To introduce the S225A substitution, the VesC Fwd primer and VesC S225A Rev primer and the VesC S225A Fwd primer and the VesC Rev primer were used to amplify each half of the *vesC* gene and introduce the mutation, and these products were then used as the template for a third PCR with the VesC Fwd and Rev primers. All cloning was confirmed using Sanger sequencing of PCR products and plasmids.

### Analysis of growth.

Comparisons of growth rates of the WT TRH7000 and Δ*epsG1*, Δ*epsL*, and Δ*epsM* mutant strains were performed using a Bioscreen growth curve analyzer (Growth Curves USA). Overnight stationary-phase cultures of V. cholerae were back-diluted as described in the figure legends and inoculated into microtiter Bioscreen plates in duplicate wells per sample. The optical density (*A*_600_) was measured at 15-min intervals for 20 h. Experiments were repeated in triplicate, and means are displayed. Growth rate analysis of WT TRH7000 containing the pVesC plasmid and the Δ*epsG1* mutant containing either pVesC or pVesC-S225A was done manually in larger flasks with good aeration, starting with overnight stationary-phase cultures that were back-diluted and then split into two cultures, where one received IPTG to a final concentration of 50 μM. The optical density (*A*_600_) was measured at 30-min intervals for 5 h.

### Genome sequencing and analysis.

Genomic DNA was isolated from V. cholerae using Wizard genomic DNA purification kits (Promega). Genomic DNA library preparation and sequencing were performed by the University of Michigan DNA Sequencing Core using Illumina HiSeq 2000. Paired-end libraries were constructed, and sequencing was performed with a read length of 100 by 100. Analysis was conducted using SeqMan software (Lasergene) for SNP and structural variant calling. Using SeqMan NGen, the TRH7000 sequence was aligned to the N16961 published reference sequence to serve as a template for analysis of T2SS mutant sequences. Variants were called using SeqMan Pro software (Lasergene), and visualization and coverage analysis were performed simultaneously. Genome sequences of T2SS mutants were compared to that of N16961 using SeqMan NGen reference-guided alignment. Variant calls that were found when comparing TRH7000 to N16961 were subtracted from the T2SS mutant calls.

### Protease secretion assay.

Extracellular protease activity was measured and quantified as described previously (19). Briefly, culture supernatants were separated from cells, and the fluorogenic probe, *N*-tert-butoxy-carbonyl-Gln-Ala-Arg-7-amido-4-methylcoumarin (Sigma-Aldrich), was added to the supernatants. Over the course of 10 min, protease activity was measured every minute using fluorescence at excitation and emission wavelengths of 385 and 440 nm, respectively. Assays were performed at least in triplicate, and values were normalized to the density of the culture (*A*_600_). Means and standard deviations (SD) are displayed.

### VesC expression and purification for crystallization.

The gene encoding residues 23 to 522 of V. cholerae VesC was cloned into a modified pRSF-Duet1 vector (Novagen) for expression with a tobacco etch virus (TEV) protease recognition site prior to the C-terminal His_6_ tag. The wild-type VesC expression was toxic for E. coli; therefore, the catalytic site residue Ser225 was replaced with Ala using the QuikChange mutagenesis protocol (Stratagene) to overcome difficulties of overexpressing VesC. To increase the expression level and solubility of the VesC (S225A) protein, a maltose-binding protein (MBP) tag followed by a TEV protease cleavage site was fused to the N terminus of VesC. The resultant plasmid containing the gene for the MBP-TEV site-VesC (S225A)-TEV site-hexahistidine tag was transformed into E. coli Rosetta2(DE3) cells. Transformed cells were grown to an *A*_600_ of ∼0.6 at 37°C in Luria broth and induced with 0.5 mM IPTG at 18°C for 4 h. The cells were harvested by centrifugation and resuspended in buffer containing 20 mM Tris-HCl, pH 8.0, 0.5 M NaCl, 2 mM FeSO_4_, 1 mM phenylmethylsulfonyl fluoride, 2 mM benzamidine, and 15 mM imidazole. Cells were lysed by sonication on ice. Soluble proteins were separated by centrifugation (30 min, 60,000 × *g*, 4°C) from the cell pellet. The protein in the supernatant was purified on a nickel-nitrilotriacetic acid (Ni-NTA) column using 150 mM imidazole, treated with TEV protease, and purified further by a second Ni-NTA affinity step, followed by anion exchange chromatography using a Source 30Q column. VesC was concentrated to 3 mg/ml and flash-frozen in liquid nitrogen.

### VesC crystallization, data collection, and structure solution.

The initial screening was performed using several commercially available sparse matrix crystallization kits with a Phoenix crystallization robot (Art Robbins Instruments), where 200-nl-volume sitting protein drops were mixed with an equivalent volume of reservoir solution. The initial crystals grew from 0.1 M Tris-HCl, pH 7.5, 0.2 M MgCl_2_, 25% polyethylene glycol (PEG) 3350 at room temperature. The optimized crystals were obtained using a crystallization solution containing 0.1 M Tris-HCl, pH 8.5, 0.2 M CaCl_2_, 0.6 M NaCl, 25% PEG 3350. The crystals were cryoprotected in the crystallization solution supplemented with 20% PEG 400 and flash-cooled in liquid nitrogen.

A 2.2-Å native data set of VesC (S225A) crystal was collected on beamline BL14-1 at the SSRL (Stanford Synchrotron Radiation Lightsource) and processed with HKL2000 (52) and *XDS* (53). The first two domains of the structure were solved by molecular replacement using PHASER (54). The initial molecular replacement solution was found by using PHASER with VesB^PD^ and VesB^Ig^ (PDB entry 4LK4; reference 41) as a search model for the protease domain and Ig fold domain of VesC. Each domain sequence was aligned with VesC and prepared for molecular replacement searching models with CHAINSAW (55).

For the C-terminal domain, molecular replacement searches were carried out using 30 templates identified by HHsearch (56), with no molecular replacement solutions found by PHASER with a TFZ score greater than 8. However, two templates, the CBM of Clostridium thermocellum (PDB entry 1UXX) and the human receptor protein tyrosine phosphatase mu (PDB entry 2C9A), gave solutions with similar orientations with a TFZ around 7. The resulting density maps, however, were uninterpretable in these regions, and it was not possible to manually improve the models.

Rosetta-based homology modeling was then carried out (57), combining pieces from these two homologous structures and refining with the Rosetta all-atom energy function (58) augmented with a term assessing agreement to density (38). After rephasing with the resulting low-energy models, the density was readily interpretable, with *Phenix* autobuild (59) building most residues in the structure, with an *R*_free_ of around 37%. Subsequent rounds of refinement in Rosetta and *Phenix* autobuild further improved the model.

The structural model was subsequently improved and completed using the program *Buccaneer* (60) and *Coot* (61) and refined with the program *REFMAC5* (62) with 8 translation/libration/screw (TLS) groups identified by the TLSMD server (63). The quality of the crystal structure was analyzed using MolProbity (64). Crystallographic data collection and refinement statistics are shown in Table 6. Least-squares analysis to determine the structural similarity was carried out using LSQKAB (65) and DaliLite (66). Protein quaternary-structure analysis was performed with the PISA server (67). The sequence alignment figure was made with ESPript (68). All other figures of molecular structures were prepared using PyMOL (DeLano Scientific Research LLC).

### Data availability.

The sequence reads from TRH7000 and the mutants were uploaded (submission ID SUB8079245) as BioProject no. PRJNA661062 to the BioProject database at NCBI. Coordinates and structure factors for the crystal structure of VesC have been deposited with the Protein Data Bank under accession code 6BQM.
