# Supplementary figures and images for: Suppressor Mutations in Type II Secretion Mutants of Vibrio cholerae: Inactivation of the VesC Protease
Source: mSphere. 2020 Dec 16;5(6):e01125-20. doi: 10.1128/mSphere.01125-20 (PMC7771236; doi:10.1128/mSphere.01125-20)

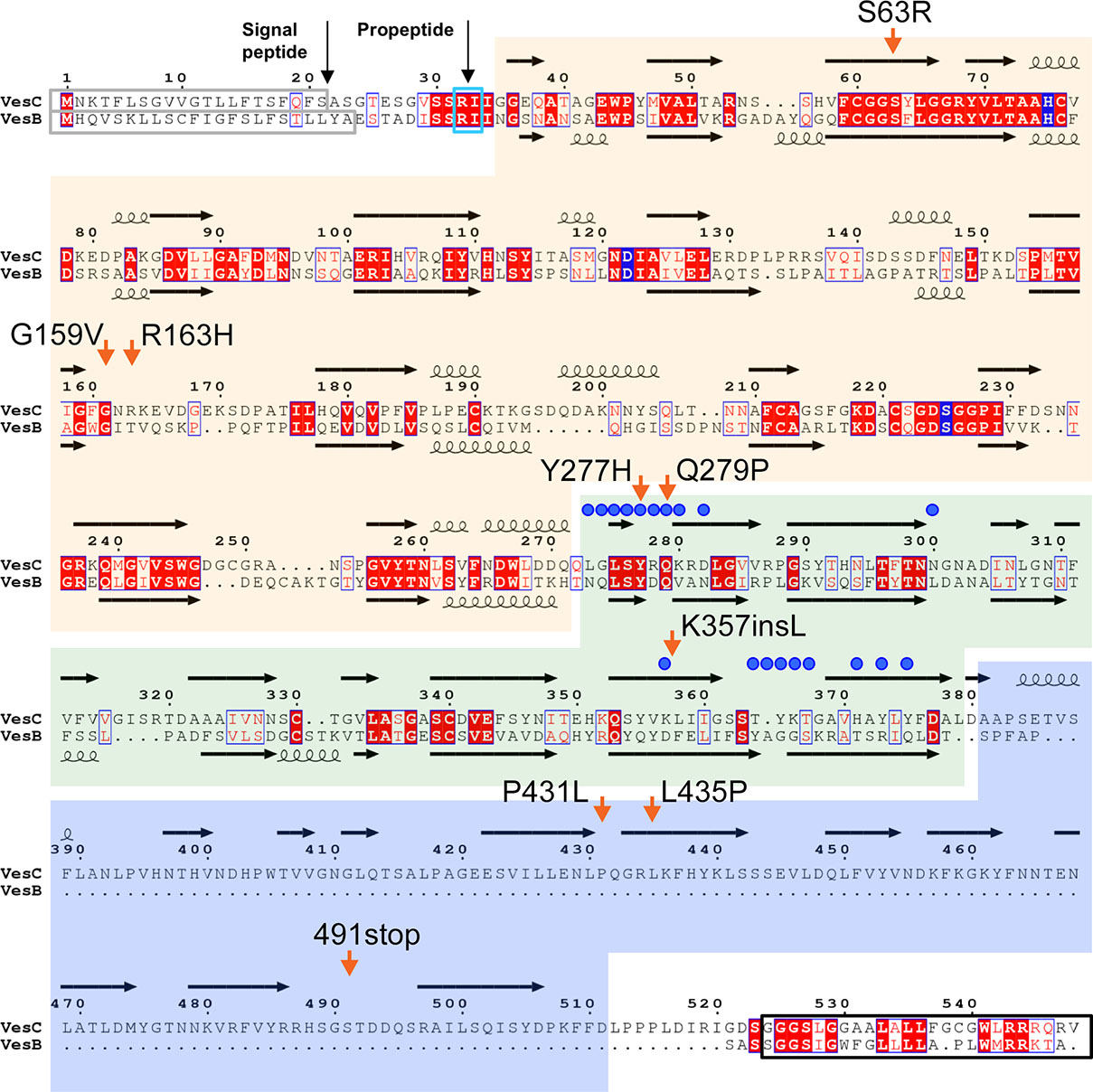

Supplement: FIG S1 [file mSphere.01125-20-sf001.jpg]

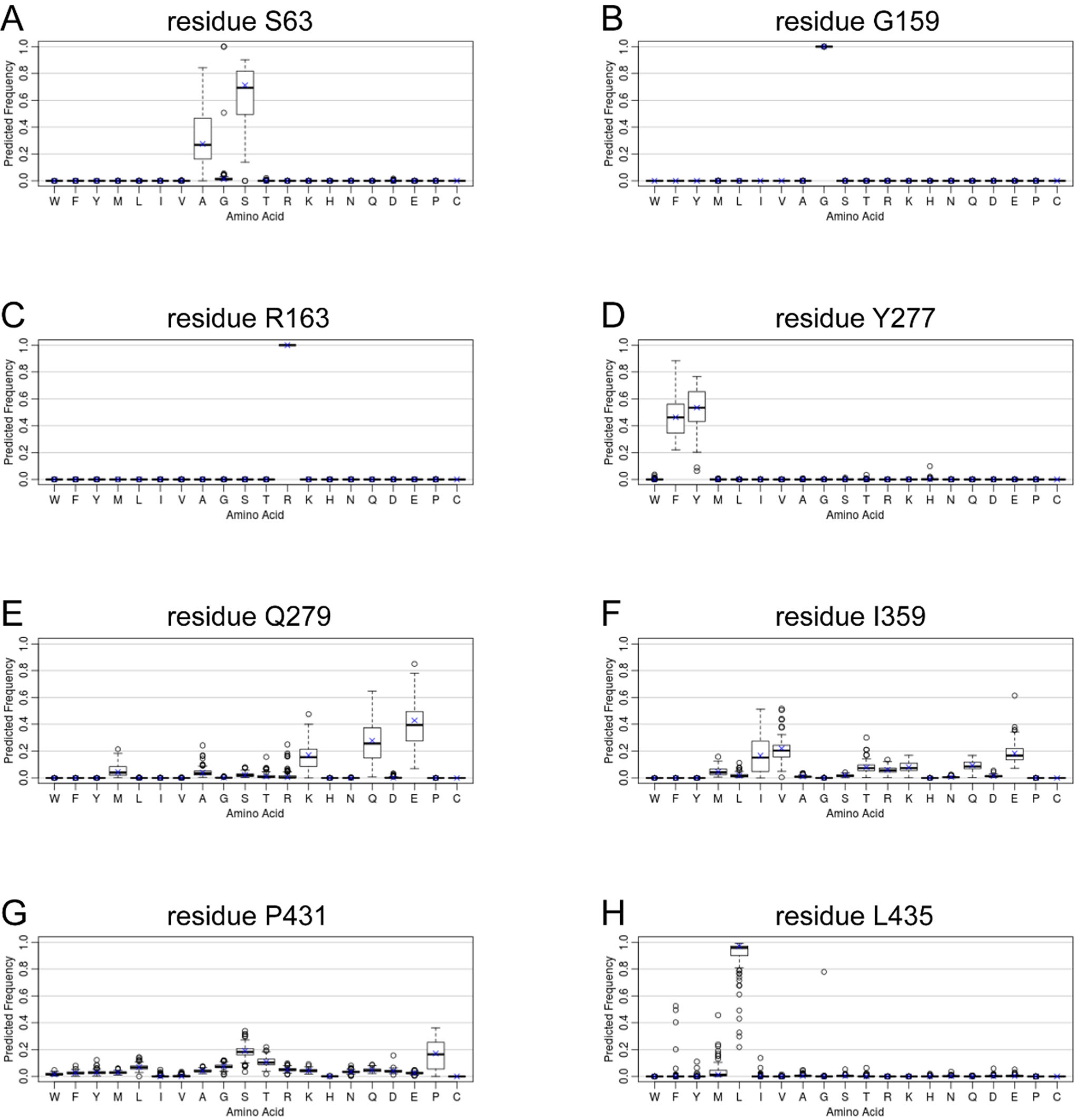

Supplement: FIG S2 [file mSphere.01125-20-sf002.jpg]

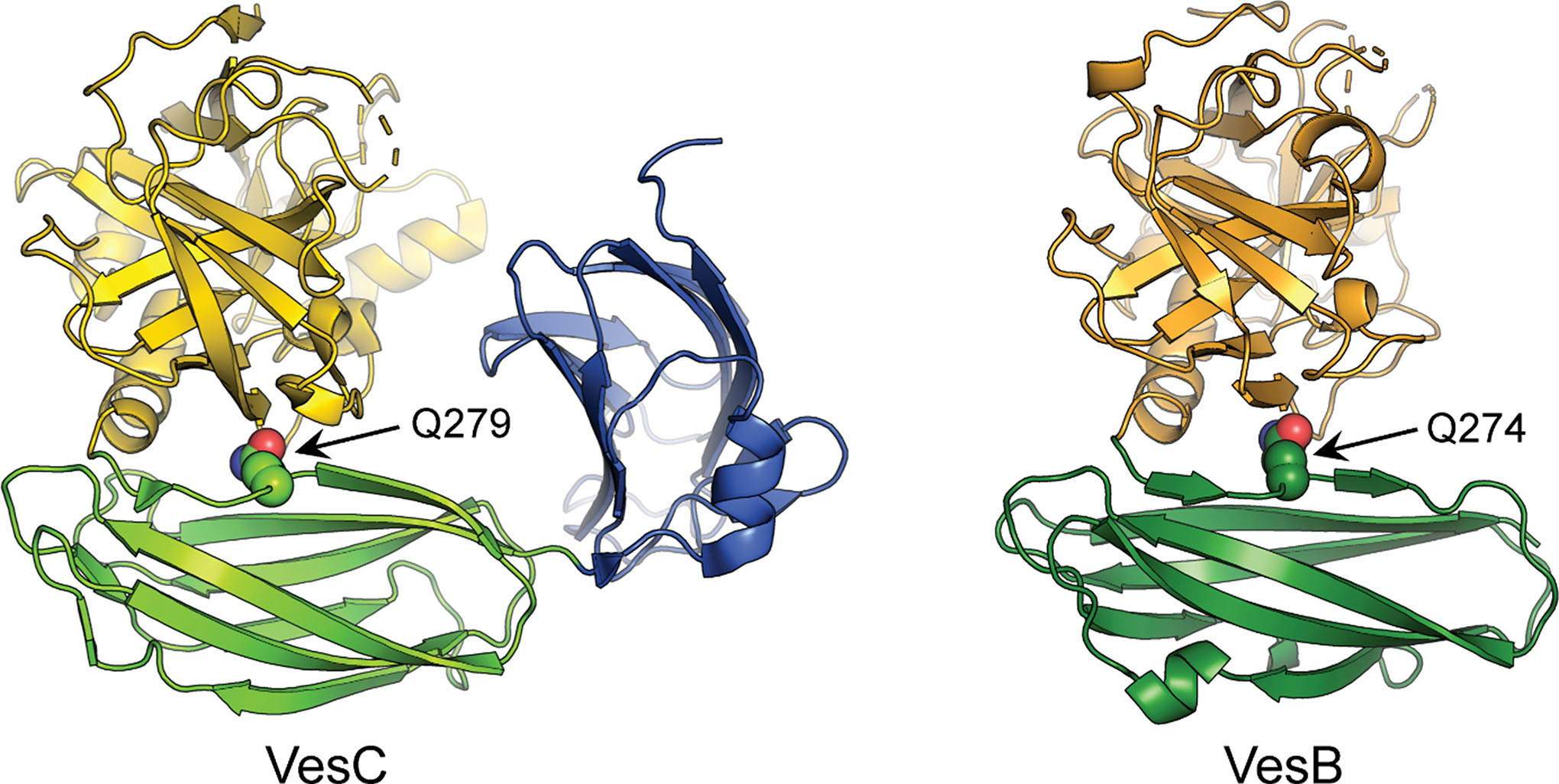

Supplement: FIG S3 [file mSphere.01125-20-sf003.jpg]
